# Cost-effectiveness evaluation of the 45-49 year old health check versus usual care in Australian general practice: A modelling study

**DOI:** 10.1371/journal.pone.0207110

**Published:** 2018-11-09

**Authors:** Si Si, John Moss, Jonathan Karnon, Nigel Stocks

**Affiliations:** 1 School of Public Health, Curtin University, Perth, Western Australia, Australia; 2 School of Public Health and Preventive Medicine, Monash University, Melbourne, Australia; 3 School of Public Health, University of Adelaide, Adelaide, Australia; 4 Discipline of General Practice, Adelaide Medical School, University of Adelaide, Adelaide, Australia; University of Groningen, NETHERLANDS

## Abstract

**Objectives:**

To assess the potential cost-effectiveness of the 45–49 year old health check versus usual care in Australian general practice using secondary data sources.

**Method:**

Risk factor profiles were generated for a hypothetical Australian cohort using data from the National Health Survey. Intervention effects were modelled based on a meta-analysis on risk factor changes in the 5 years after a health check. The Framingham Risk Equation was applied to estimate the 5-year cardiovascular disease (CVD) incidence in the health check and usual care group respectively. A Markov model was then constructed to extrapolate long-term CVD outcomes, health care costs and Quality Adjusted Life Years (QALYs) in both groups. Health check-related costs, applied to the health check group, were estimated from clinical guideline and experts’ opinion. Lifetime costs, applied to both groups, included costs of hospitalization for CVD events and associated post-event health service use. The Incremental Cost-Effectiveness Ratio (ICER) was calculated for male and female patients respectively.

**Results:**

Compared to usual care, the health check reduced CVD incidence for both males (RR = 0.87) and females (RR = 0.91) over a 5-year time. In a lifetime projection, health check led to an average 0.008 and 0.003 QALYs gained per male and female participants respectively. The estimates ICERs were AU $42,355 and AU $133,504 per QALY gained for males and females, respectively. A probabilistic sensitivity analysis demonstrated a probability of cost-effectiveness of 17.5% and 0% for male and female attendees, assuming a willingness to pay threshold of AU $28,000 per QALY gained.

**Conclusion:**

The 45–49 year old health check is associated with a small expected QALY gain per participant, though the persons avoiding CVD events experience large health gains. The mean ICER is larger than an empirical estimate of the threshold ICER and the evaluated health check is highly unlikely to be cost-effective.

## Introduction

Government funded health check programs have recently been initiated in several countries including England [1] and Australia [2], but the effectiveness of such programs compared to usual care (no health check) has been questioned. Two systematic reviews reported no significant benefits in total mortality after health checks [3, 4]. However, one of them reported significant improvements in surrogate outcomes (i.e. blood pressure, total cholesterol BMI and smoking cessation) after general practice-based health checks [3]. Even though it is biologically plausible that improved control of risk factors should prevent or delay the onset of chronic disease and improve patient’s Quality of Life (QOL), very few studies have reported morbidity and QOL changes after health checks [3]. Therefore, modelling studies have been widely used to bridge the gap and to predict the cost-effectiveness of such programs [5–7].

In Australia, a 45–49 year old health check was introduced in 2006. This Medicare funded ‘well person’ check-up targets patients with at least one identifiable risk factor (lifestyle, biomedical or family history) [2]. The objective was to detect early stage chronic disease or risk factors and promote healthy lifestyles. Unlike other government funded health checks, this program is a one-off check-up for cardiovascular risk factors. No further subsidized follow-up or intervention programs are specified by Medicare, except for the routine care of identified risk factors. Pathology tests are ordered if deemed necessary by General Practitioners (GPs). Completion of a health check usually takes another 1–2 practice visit(s) depending on the identified risk factors and whether pathology results need to be reviewed [8]. Follow-up interventions including further consultations, medications or referrals are indicated if risk factors are detected. Currently, Medicare Australia employs a time-dependent payment mechanism to reimburse this program.

There has been a lack of evidence on the effectiveness of this health check program compared to usual care and its economic impact on the health care system. The National Institute for Health and Clinical Excellence (NICE) in the UK has set a threshold of £20,000 per QALY for health service to be considered cost-effective compared to their best alternative [9]. Although there has not been an official statement about a threshold value for health services in Australia, a number of Australian policy reviews have suggested that a medication [10, 11] or preventive health service [12] with estimated willingness to pay threshold of AU $50,000 per QALY compared to their best alternative is more likely to be approved and funded [10, 11]. However, a more recent study investigated the association between incremental government health expenditure and mortality-related QALY gained in general population as imperial reference to the Incremental Cost Effectiveness Ratio (ICER) for new health technologies in Australia. The study reported ICER at around AU $28,000 per QALY gained [13]. The aim of this study was to assess the cost-effectiveness of the 45–49 year old health check compared to usual care in Australian general practice using secondary data sources, and to inform the need for primary data collection for further evaluation.

## Methods

### Model structure

**Fig 1:**
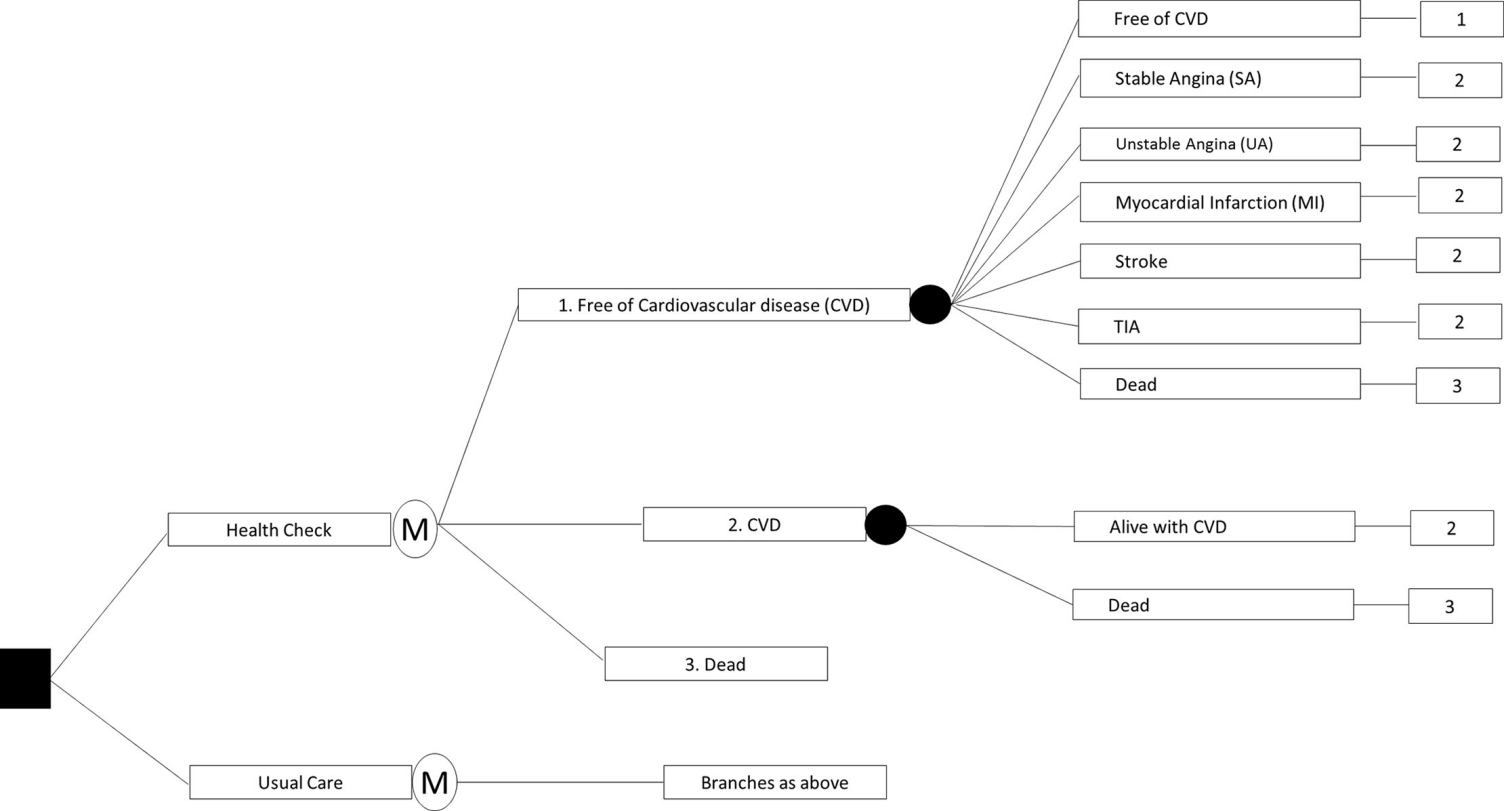
Model structure.

A decision analytic model was developed comparing the cost-effectiveness of the health check versus usual care. The model comprised seven health states: ‘alive without cardiovascular disease (CVD)’, ‘alive stable angina (SA)’, ‘alive with unstable angina (UA)’, ‘alive with myocardial infarction (MI)’, ‘alive with stroke’, ‘alive with transient ischaemic attack (TIA)’, and ‘dead’; as well as five transition states marking the first event of SA, UA, MI, stroke and TIA. The cycle length was one year (Fig 1). The analysis was undertaken from the perspective of the Australian health care system (both federal and state government), and a discount rate of 5% per annum was applied.

There is insufficient evidence suggesting causal effect (relative risks) of this health check program on reducing long-term CVD events, but evidence on potential benefits in risk factor modifications (3). Therefore, we applied Framingham Risk Equation (FRE) to translate risk factors into predicted risks of CVD events with and without health checks. We apply the predicted CVD incidence as transition probabilities (in the first 5 cycles) in both the health check and usual care group in a Markov model.

### Model population

The model population comprised ‘healthy’ Australians eligible for the 45–49 years health check program. Follow-up was simulated until death or the end of 50 yearly cycles since entry to the cohort. All subjects started in the simulation in the health state ‘Alive, no CVD’.

### Model inputs

We constructed a hypothetical cohort of 10,000 Australians aged 45–49 years (5,000 males and 5,000 females). The age- and gender- specific proportional distributions of risk factors (systolic blood pressure, total cholesterol, HDL and smoking status) in the target population were derived from the 2011Australian National Health Survey (ANHS) (S1 Table) [14]. Each individual in the hypothetical cohort was randomly assigned a set of risk factors based on their respective distributions.

### Treatment effects

To model the effectiveness of health check, we translated the improvement of risk factor control into decrease in CVD incidence using the FRE (5-year risk equation). We assume no benefit from health check beyond 5 years. Using data from a published meta-analysis of general practice-based health check studies [3], intervention effects were modelled by applying Relative Risk (RR) of patients remaining at high risk after intervention to the following risk factors: systolic blood pressure (SBP>140mmHg), total cholesterol (TC>6mmol/L) and smoking (Table 1). By applying the RR to the proportion of patients with risk factors above the thresholds, we are able to re-allocate high-risk patients into low risk groups for population in the health check group.

**Table 1:** Model inputs.

*Variable*	*Descriptions*		*Value*	*Distributions*
**Risk reduction after health check (RR)**	High TC (TC>6mmol/L)		0.63 (0.50, 0.79)	Lognormal
	High SBP (SBP>140mmHg)		0.71 (0.55, 0.9)	
	Smoking (Current smoker)		0.90 (0.84,0.97)	
**CVD events allocation**	45–49 years	Male	UA:22.19%; SA:17.05%; MI:42.87%; Stroke:11.37%; TIA:1.91%	Uniform
		Female	UA:26.47%; SA:16.89%; MI:26.73%; Stroke:20.39%;TIA:6.99%	
**Annual incidence**	50–54 years	Male	UA: 0.20%; SA: 0.16%; MI: 0.40%; Stroke: 0.10%; MIA: 0.02%	
		Female	UA: 0.10%; SA: 0.06%; MI: 0.10%; Stroke: 0.08%; MIA: 0.03%	
	55–64 years	Male	UA: 0.45%; SA: 0.41%; MI: 0.70%; Stroke: 0.24%; MIA: 0.05%	Uniform
		Female	UA: 0.19%; SA: 0.16%; MI: 0.22%; Stroke: 0.13%; MIA: 0.04%	
	65–74 years	Male	UA: 0.75%; SA: 0.78%; MI: 1.04%; Stroke: 0.56%; MIA: 0.13%	
		Female	UA: 0.37%; SA: 0.34%; MI: 0.46%; Stroke: 0.33%; MIA: 0.07%	
	75–84 years	Male	UA: 1.04%; SA: 0.95%; MI: 1.66%; Stroke: 1.29%; MIA: 0.21%	
		Female	UA: 0.67%; SA: 0.58%; MI: 1.07%; Stroke: 1.01%; MIA: 0.10%	
	85–94 years	Male	UA: 0.99%; SA: 0.81%; MI: 2.50%; Stroke: 2.09%; MIA: 0.16%	
		Female	UA: 0.63%; SA: 0.59%; MI: 1.72%; Stroke: 2.00%; MIA: 0.08%	
**Mortality**	45–54 years		Male: 0.277%; Female: 0.176%	Uniform
	55–64 years		Male : 0.658%; Female : 0.378%	
	65–74 years		Male : 1.628%; Female : 0.990%	
	75–84 years		Male : 5.004%; Female : 3.292%	
	85–94 years		Male : 11.803%; Female : 11.257%	
**SMR** ***Mean (95% CI)***	UA		2.19 (2.05; 2.33)	
	SA		1.95 (1.65; 2.31)	Lognormal
	TIA		1.4 (1.1; 1.8)	
	MI		Male : 2.28 (2.12; 2.46); Female : 3.07 (2.70; 3.48)	
	Stroke		Male : 2.58 (2.43; 2.75); Female : 2.85 (2.66; 3.05)	
**Population Utility weights** ***Mean(SE)***	40–49 years		Male : 0.84 (0.19); Female : 0.86 (0.17)	Beta
	50–59 years		Male : 0.82 (0.20); Female : 0.79 (0.23)	
	60–69 years		Male : 0.80 (0.18); Female : 0.77 (0.21)	
	70–79 years		Male : 0.79 (0.22); Female : 0.72 (0.26)	
	80+ years		Male : 0.71 (0.30); Female : 0.63 (0.28)	
**CVD Utility weights** ***Mean(SE)***	UA		0.770 (0.038)	Beta
	SA		0.808 (0.038)	
	MI		0.760 (0.018)	
	Stroke		0.629 (0.04)	
	TIA		1	
**Health check-related costs *Mean(range)***	Male		$393 ($193, $660)	Uniform
	Female		$355 ($193, $589)	
**Hospitalization Costs**	UA		$2,682 (±25%)	Uniform
	SA		$2,146 (±25%)		
	MI		$5,572 (±25%)	
	Stroke		$6,496 (±25%)	
	TIA		$3,128 (±25%)	

Using the generated risk factor profiles, individual five-year CVD risk were calculated using the Framingham Risk Equation (FRE) [15]. The average of CVD risks of 5000 males and females respectively were used to estimate CVD incidence in the target population. We then repeated the simulation 1000 times. In the usual care arm, we directly applied the population risk distributions from the NHS. In the health check arm, we derived a new set of risk factor distributions by applying the risk factor modification effects to the population risk distributions. The estimated event risks from the usual care group were later validated against CVD hospitalization data from the National Hospitalization Morbidity Database (NHMD) [16]. We then summarize the mean and 95% credibility intervals of CVD risks from the 1000 simulations in both the control and intervention arms.

The simulated annual risks of CVD were then allocated to MI, UA, SA, TIA and stroke to represent incidences of the five CVD sub-states. Using data from the NHMD, we calculate the proportions of UA, SA, MI, stroke and TIA out of all CVD hospitalization episodes respectively in a year using the following formula [16].

$$P(UA)=\frac{No.(UA)}{No.(UA+SA+MI+stroke+TIA)}$$

Since we assume 5-year effects, from year six onwards, same transition probabilities were applied to the Markov model in both groups. Age and gender specific CVD free mortality rates were derived from the 2011 national cause of death report [17]. Standardised Mortality Ratios (SMRs) were multiplicatively applied to the population mortality data to estimate transition probabilities to death from CVD sub-states [18–22]. The key model inputs are summarised in Table 1.

### Costs

We then estimated the direct costs associated with the health check by applying guideline recommended interventions (GP visits, pathology tests and medications) to those health check-detected high risk patients. Based on current clinical guidelines and experts’ opinions on CVD risk factor management, we estimated the annual service use related to the following risk conditions: smoking, hypertension (mild or moderate and severe systolic high blood pressure), high total cholesterol that requires medications, and high TC combined with systolic hypertension. The details of services use and unit costs are summarised in S2 Table. The average annual costs per patients were calculated by multiplicatively applying the unit intervention cost to the prevalence of risk conditions and treatment uptake and compliance rates derived from literature review [5]. We further estimated the minimum cost of health check assuming no further follow-up interventions and maximum cost of health check assuming full treatment uptake and compliance following health check. We assume no cost in primary health care for CVD in the control group.

Besides direct costs associated with the health check (as described above), the costs of long-term health service use in both health check and usual care groups were included. The long-term costs for health service use include acute event cost, extracted from the National Hospital Cost Data Collection (NHCDC) database [23], and associated post-CVD state costs. The costs of post-CVD states were estimated to be 15% of the acute event costs [18, 24]. The inputs are summarised in Table 1.

### Utilities

The age- and gender- specific utilities for general population and the disutility weights for acute CVD events were derived from literature review [25, 26]. We further assumed reduced disutility weights (50% reduction in disutility weights of acute events) post-event in the base-case scenario.

### Scenario analysis

Scenario analyses were applied using the following parameters:

- Reduced disutility for post-CVD states: 0%, 25%, 75% and 100% reduction in disutility weights of acute event disutility weights
- The costs of post-CVD states were estimated to be 10% and 20% of the acute event costs.
- Alternative annual discount rate of 3.5% was applied.

### Sensitivity analysis

A probabilistic sensitivity analysis (PSA) was undertaken to represent the combined effects of uncertainties across all input parameters in the Markov model. Probability distributions were constructed to represent the uncertainties around input variables in the Markov model, including the incidence of CVD in the both arms, SMRs, utility decrements for the post-CVD states and costs for CVD hospitalizations (Table 1). We ran the Markov model 1,000 times. The mean values and 95% credibility intervals of LYs and QALYs were summarized. The cost-effectiveness planes (CEP) and cost-effectiveness acceptability curves (CEAC) were presented by sex separately.

## Results

### Short term outcomes and direct costs for health check

The simulated 5-year CVD incidence of 10,000 individuals (5,000 males and females respectively) aged 45–49 years in the usual care group was validated against national CVD incidence statistics (S3 Table). The 5-year event rates from1,000 cohort simulation repetitions of the target population and range of direct costs associated with health check are summarized in Table 2.

**Table 2:** 5-year CVD incidence rates in the health check and usual care groups and direct costs for health checks.

*CVD risks*	*Usual care (%)*	*Health check (%)*	*Diff*.*(%)	RR**	*Direct cost of health check*
	Mean (2.5 & 97.5 percentiles)				Mean (Min, Max)
Males	3.7 (3.7, 3.8)	3.3 (3.2, 3.4)	0.5 (0.3,0.7)	0.87 (0.8, 0.9)	$393 ($193, $658)
Females	1.5 (1.4, 1.5)	1.3 (1.3, 1.4)	0.1 (0.1, 0.2)	0.9 (0.8, 0.9)	$355 ($193, $587)

For each cohort simulation

*Diff. = CVD incidence_intervention_ -CVD incidence_control_

**RR = CVD incidence_intervention_ /CVD incidence_control_

Estimates made from 1,000 repetitions of cohort simulation

CVD incidence decreased 0.49% in five years after the health check (with RR = 0.87). The health check was less effective among females due to their lower baseline risks, with only 0.14% CVD events were avoided in five years (RR = 0.91).

### Base-case cost-effectiveness analysis

Under the base-case scenario, the health check was associated with 0.019 LYs or 0.008 QALYs gained per male attendee over 50 years. The estimated ICER were AU$18,483 per LY gained and AU $42,355 per QALY gained. For every female attendee, the estimated LYs and QALYs gained were 0.007 LYs and 0.003 QALYs with ICERs of AU $51,450 per LY gained and AU $133,504 per QALY gained (Table 3).

**Table 3:** Lifetime health outcome and medical cost of the 45–49 year health check.

		*Control*	*Intervention*	*Diff*.	*$/LYs Gained*	*$/QALY Gained*
**Male**	LY	33.364	33.384	0.019		
	QALY	12.409	12.417	0.008	**$18,483**	**$42,355**
	Cost	$1,753	$2,109	$356		
**Female**	LY	37.423	37.430	0.007		
	QALY	12.759	12.762	0.003	**$51,450**	**$133,504**
	Cost	$1,018	$1,361	$343		

Assumptions: 50% reduction in disutility of acute events applied for post-CVD states; 15% of acute event costs for post-CVD states

### Scenario analysis

The results of scenario analyses are summarised in Table 4. The ICERs per QALY gained were sensitive to the utility weights compared to the annual costs assigned to post-CVD states.

**Table 4:** Results of scenario analysis for the 45–49 year health check vs usual care.

Scenarios		Diff. YLL	Diff. QALY	$/YoLS	$/QALY
**Base case**	Males	0.019	0.008	$18,483	$42,355
	Females	0.007	0.003	$51,450	$133,504
**Disutility weight for post-CVD states**					
0 reduction in disutility post-acute event	Males	0.019	0.013	$18,483	$18,483
	Females	0.007	0.004	$51,450	$51,450
25% reduction in disutility post-acute event	Males	0.019	0.011	$18,483	$33,093
	Females	0.007	0.003	$51,450	$104,096
75% reduction in disutility post-acute event	Males	0.019	0.006	$18,483	$58,816
	Females	0.007	0.002	$51,450	$186,067
100% reduction in disutility post-acute event	Males	0.019	0.004	$18,483	$96,205
	Females	0.007	0.001	$51,450	$306,904
**Annual costs for post-CVD states**					
10% of acute event costs	Males	0.019	0.008	$19,009	$43,560
	Females	0.007	0.003	$51,937	$134,768
20% of acute event costs	Males	0.019	0.008	$17,957	$41,149
	Females	0.007	0.003	$50,963	$132,239
**Annual discount rate**					
3.5% annual discount	Males	0.019	0.011	$18,176	$31,684
	Females	0.007	0.003	$51,124	$99,703

### PSA

**Fig 2:**
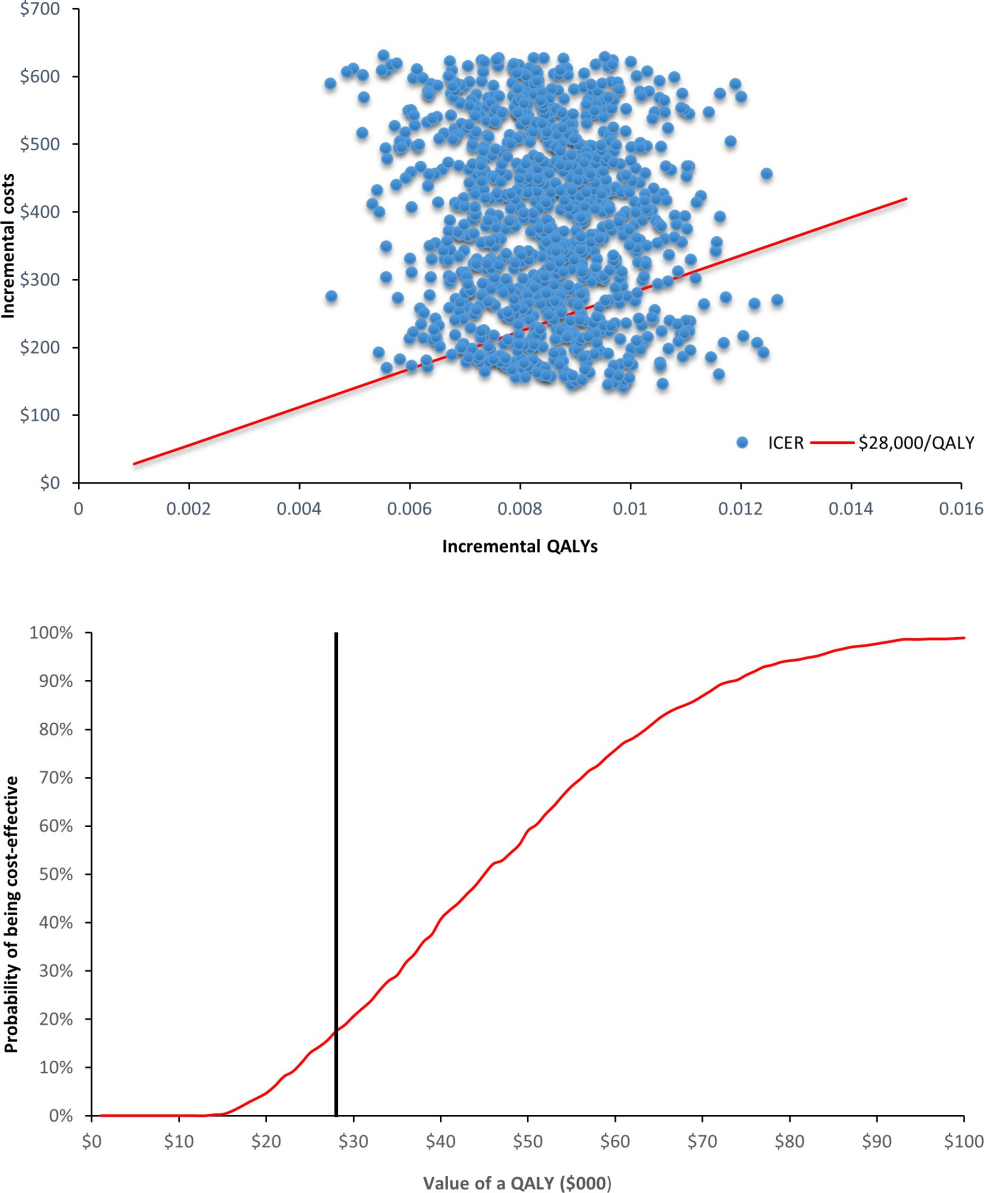
CEP & CEAC of health check versus usual care in males.

**Fig 3:**
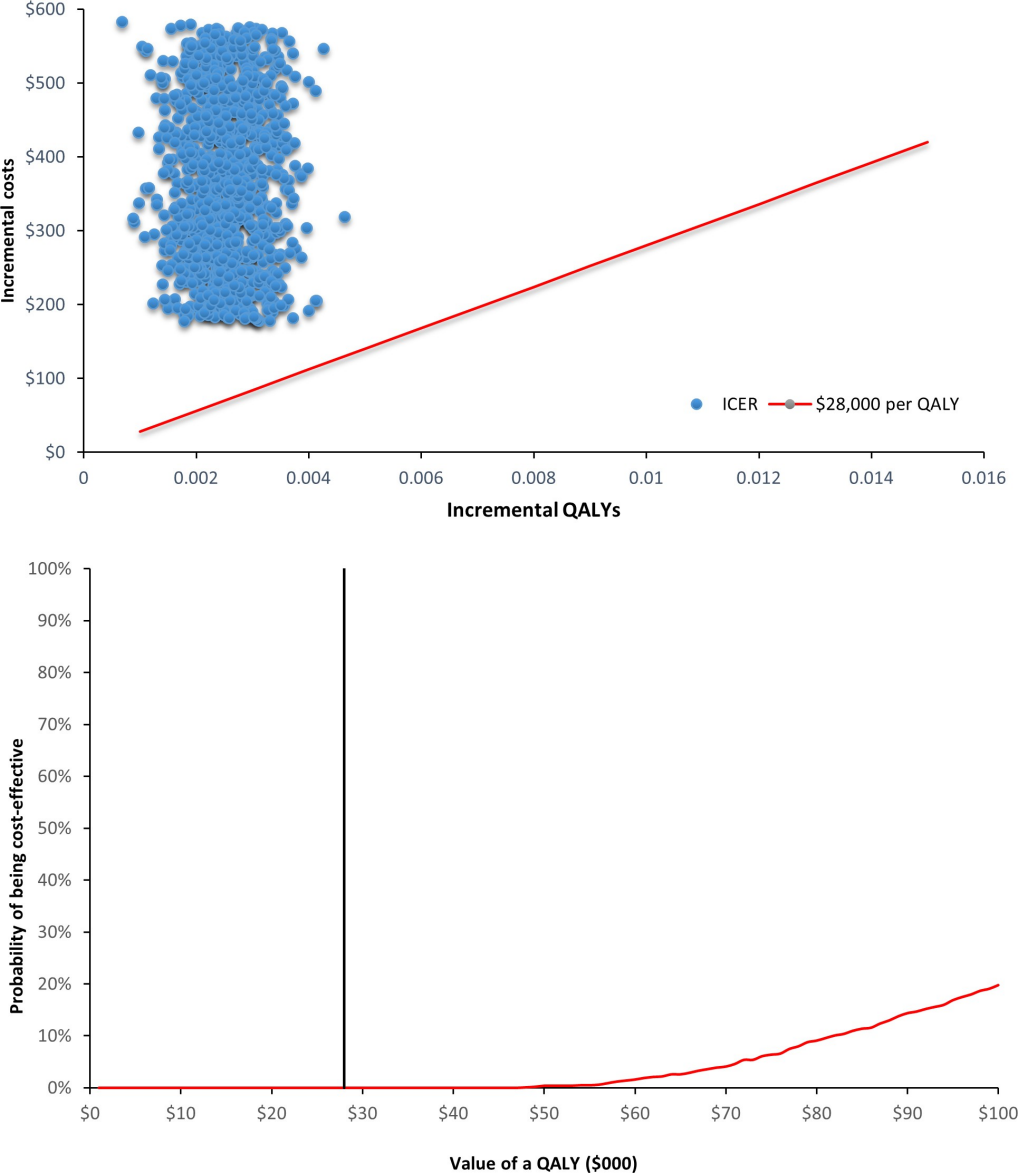
CEP & CEAC of health check versus usual care in females.

Under the base-case scenario, the probability of cost-effectiveness under the willingness to pay threshold of AU $50,000/$28,000 per QALY was 59.0%/17.5% for male participants (Fig 2). For female participants, the probability of cost-effectiveness under the willingness to pay threshold of AU $50,000/$28,000 per QALY was 0.4%/0% for female participants (Fig 3).

## Discussion

In this study, we applied estimated RRs of individuals in the general population remaining at high risk for a range of factors (systolic blood pressure, cholesterol, and smoking status) following a health check at age 45–49 years [3]. Predicted 5 year CVD incidence reduced by 13% in males (RR_males_ = 0.87) and 9% in females (RR_females_ = 0.91) after the intervention, which is comparable to cohort studies that reported a RR of CVD around 0.9 after a health check [27, 28]. The estimated incremental cost-effectiveness of the 45–49 year old health check program was AU $42,355 per QALY and AU $133,504 per QALY gained for male and female attendees respectively. The results indicated that the 45–49 years health check program is unlikely to be cost-effective relative to other funded health technologies.

We found trivial QALY improvement for the 45–49 year old health check in both males and females. Despite the ICER per QALY gained for males was largely under the rule of thumb willingness to pay threshold of AU $50,000 per QALY gained, it is inappropriate to claim cost-effectiveness. It is well recognized that the public preferences for QALYs differed by the causes and severity of diseases [29]. Compared to life-threatening conditions and those largely attributable to medical treatments, people then to place lower weight to conditions that could be improved by primary health services and non-medical treatment (e.g. lifestyle modifications) [29]. Therefore, more conservative ICER threshold should be used to assess the cost-effectiveness of primary health services including health checks. Our estimated ICERs per QALY gained of this health check for both male and female attendees were way above the proposed new threshold of AU $28,000 per QALY gained for preventive health services [13]. Thus, the 45–49 year old health check is unlikely to be cost-effective, especially for female participants.

Previous studies have assessed the cost-effectiveness of health check programs versus usual care in six European countries [5, 6, 30, 31] and Australia [7]. These studies simulated regular health checks (5 yearly) for patients aged 40–75 years with no pre-existing CVD or diabetes; subsequent interventions were specified to manage detected risk factors [32]. Three of these modelling studies adopted Cost-Effectiveness Analysis (CEA) [5–7] and the other two were Cost-Consequence Analysis (CCA) [29, 30]. All of them used micro-simulation to predict individual risk of chronic disease. Effectiveness of the health check programs were estimated by simulating the screening and intervention procedures incorporating detection rates; intervention rates; compliance rates and the potential effectiveness of specific interventions (e.g. smoking cessation; prescriptions for anti-hypertensive, statins and lifestyle interventions). Three CEA studies concluded that five yearly health checks to the 40–75 year olds were likely to be cost-effective or cost saving in six European countries (the UK, Denmark, France, Germany, Poland and Italy) and Australia [5–7]. Furthermore, both CCA studies concluded that targeted screening of high risk or socio-economically deprived groups were more effective than universal screening [30, 31].

In comparison to these studies, our modelling study was designed to investigate the cost-effectiveness of a one-off 45–49 year old health check program in Australian general practice. Methodologically, we constructed a Markov model to simulate CVD incidence and mortality in a fixed cohort. Furthermore, given the lack of empirical data on interventions subsequent to a health check program, rather than simulating the screening and intervention processes, we directly applied surrogate outcome effects (derived from a systematic review) to the simulated baseline risk factor prevalence rates to estimate the benefits of a health check.

To predict CVD incidence in the baseline cohort, the Framingham risk equation (FRE) was applied to individuals in the simulated hypothetical cohorts. Acknowledging concerns about the generalizability of the FRE, and its applicability to all CVD outcomes [32], we carefully considered the applicability of the FRE to our study. The application of the FRE to Australian cohorts has been validated and recommended in clinical practice [33, 34]. The target population was healthy persons with no pre-existing chronic disease of any kind, which is consistent with the Framingham cohort. Furthermore, the model outputs were validated against national CVD hospitalization data (S3 Table). While the model presented reasonable estimates of CHD incidence, it consistently underestimated CVA incidence. It has been argued that the FRE was initially developed to predict CHD risk rather than other forms of CVD [32]. However, the influence was not substantial given the generally low CVA incidence in the target population (0.12% in male and 0.10% in female). Therefore, when combining CHD and CVA, the model presented reasonable estimates of overall CVD incidence to the national estimates (within the 95% confidence interval of CVD estimates). These results validated the simulation of cohort CVD incidence using the FRE and population-based risk factor distributions.

### Assumptions and limitations

Since it is biologically plausible to prevent or delay the onset of chronic disease through early detection and management of risk factors, we assumed the improved control of risk factors would translate into morbidity and mortality benefits in subsequent years. The reason that we apply FRE to predict CVD risks before and after health check, instead of directly applying causal effects on CVD risks, is because it is difficult to ascertain the size of causal effects of health checks on general population. Compared to clinical trials of pharmaceutical interventions, the target population of health checks are generally young and healthy, with generally low event rates. Therefore, the majority of health check trials/studies were not sufficiently powered to draw causal inferences between health checks and long-term CVD events or mortality.

It is likely that our model may have underestimated the effects of the 45–49 year old health check program due to conservative assumptions adopted in the simulation regarding the magnitude and longevity of the intervention effects. Firstly, the reported risk factor changes in the systematic review are likely to be underestimates, since most of the included trials were conducted in the 1990s, when preventive health care guidelines were relatively conservative. Since then, the clinical management of risk factors has improved with more effective pharmacotherapy (e.g. statins and anti-hypertensive drugs). Secondly, we assumed a duration of intervention effects of five years, because most trials followed up for less than 5 years. It is likely that the benefits could persist beyond 5 years. Thirdly, we did not simulate repeat CVD events in the Markov model. Since patients with a history of CVD events are more likely to suffer another CVD event, the single event assumption is likely to underestimate the cost savings and quality of life benefits of health checks. Fourthly, we only simulated potential benefits of averted CVD events in this study. However, the 45–49 year old health check program can also incorporate assessments of depression and selective screening for cancers (e.g. skin; cervical and colorectal cancer etc.) to high risk patients [2]. Therefore, this health check could potentially lead to other health benefits beyond CVD morbidity and mortality.

On the other hand, we did not consider the potential harmful effects of medical treatment on patients’ quality of life. However, in clinical practice, medications are not usually the first choice for patients with elevated risk factors, especially for these relatively young and otherwise healthy patients. Lifestyle modification is often the first recommendation. As reported in a few health check trials, no significant increase in prescription rates were observed after health checks compared to the usual care group [35]. Furthermore, the side-effects of anti-hypertensive or lipid lowering medications are generally uncommon and there are multiple alternative medicines if this does occur. Therefore, the influence of omitting such effects would not be substantial.

There also remains uncertainty around the cost estimates for the health check and associated treatment of risk factors. We estimated health check-related costs based on clinical guidelines and experts’ opinions on risk factor management, regarding ordered pathology tests, repeat GP visits, and prescribed pharmaceuticals. We note that risk factor treatment costs will also be incurred in the absence of the health check, following the opportunistic or incidental detection and treatment of risk factors. Since we assume no CVD-related primary health care costs in the control group for 5 years, the cost differences of the intervention and control group are likely to be an overestimate, which would lead to an over estimate of ICER. However, the effect is unlikely to be large, considering the relatively low prevalence of risk factors in the target population.

### Strengths

Given the limitations, our study provides estimates of the cost and effectiveness of the 45–49 year old health check program in Australian general practice. This is also the first study to investigate the economic impact of this Medicare funded program.

### Future studies

In the absence of randomised controlled trial of the health check program, the cost-effectiveness of the Australian health check program could be better informed by primary data describing the identification and treatment of risk factors for patient attending, and not attending the health check. These data would inform a significantly more complex model that extrapolates costs and outcomes from the detection and treatment of risk factors in the intervention and control groups.

### Implications

The results suggest that under the current Medicare scheme, the 45–49 year old health check program is not cost-effective in females and unlikely to be cost-effective in males. Furthermore, the lack of definable intervention protocols after the initial check-up imposes considerable uncertainties on both the effectiveness and cost estimates of this program. Guideline oriented intervention strategies for at-risk patients may improve performance.

## Supporting information

S1 Table Model inputs. (DOCX)

S2 Table Health check-related short term medical services use estimates. (DOCX)

S3 Table Model validation—Annual CVD incidence (45–54 year old Australians). (DOCX)
