# Supplementary material for: Cost-effectiveness evaluation of the 45-49 year old health check versus usual care in Australian general practice: A modelling study
Source: PLoS One. 2018 Nov 9;13(11):e0207110. doi: 10.1371/journal.pone.0207110 (PMC6226178; doi:10.1371/journal.pone.0207110)
Supplement: S1 Table — (DOCX) [file pone.0207110.s001.docx]

S1 Table: Model inputs

| **Variable** | **Subgroups** | **Value** | **Distributions** |
| --- | --- | --- | --- |

|  |  | **Males** | **Females** |  |
| --- | --- | --- | --- | --- |
| ***Population risk factor distributions*** | | | | |
| **SBP** | <100 mmHg | 1.90% | 7.40% | Fixed |
|  | ≥100 to <110 mmHg | 12.80% | 19.10% |  |
|  | ≥110 to <120 mmHg | 22.60% | 24.30% |  |
|  | ≥120 to <130 mmHg | 25.80% | 22.10% |  |
|  | ≥130 to <140 mmHg | 16.50% | 12.60% |  |
|  | ≥140 to <150 mmHg | 11.40% | 7.50% |  |
|  | ≥150 to <160 mmHg | 5.70% | 3.50% |  |
|  | ≥160 to <170 mmHg | 2.20% | 2.40% |  |
|  | ≥170 mmHg | 1.10% | 1.10% |  |
| **TC** | <4.0 mmol/L | 11.94% | 9.63% | Fixed |
|  | ≥4.0 to <4.5 mmol/L | 11.94% | 12.47% |  |
|  | ≥4.5 to <5.0 mmol/L | 15.90% | 16.66% |  |
|  | ≥5.0 to <5.5 mmol/L | 16.06% | 15.99% |  |
|  | ≥5.5 to <6.0 mmol/L | 19.22% | 20.23% |  |
|  | ≥6.0 to <6.5 mmol/L | 12.67% | 12.30% |  |
|  | ≥6.5 to <7.0 mmol/L | 7.22% | 6.56% |  |
|  | ≥7.0 mmol/L | 5.04% | 6.15% |  |
| **HDL** | <1.0 mmol/L | 19.1% | 4.86% | Fixed |
|  | ≥1.0 to <1.3 mmol/L | 41.7% | 22.69% |  |
|  | ≥1.3 to <1.5 mmol/L | 21.3% | 23.17% |  |
|  | ≥1.5 to <2.0 mmol/L | 16.0% | 38.92% |  |
|  | ≥2.0 to <2.5 mmol/L | 1.5% | 9.55% |  |
|  | ≥2.5 mmol/L | 0.3% | 0.81% |  |
| **Smoking** | Current smoker | 24.0% | 18.9% | Fixed |
