# Supplementary material for: Cost-effectiveness evaluation of the 45-49 year old health check versus usual care in Australian general practice: A modelling study
Source: PLoS One. 2018 Nov 9;13(11):e0207110. doi: 10.1371/journal.pone.0207110 (PMC6226178; doi:10.1371/journal.pone.0207110)
Supplement: S2 Table — (DOCX) [file pone.0207110.s002.docx]

S2 Table: Health check-related short term medical services use estimates

|  | **GP visits** | **Medication** | **Pathology test** | **Annual Intervention cost** | **Prevalence**  % (95%CI) | **Uptake rate** | **Annual compliance rate** |
| --- | --- | --- | --- | --- | --- | --- | --- |
| **Health check** | Standard health check + 1 GP visit |  | TC & HDL test | $192.6 | n/a | 100% | n/a |
| **Smoking** | 3 visits | NRT for 12 weeks |  | $277.8 | M: 24.0 (22.9, 25.4)  F: 18.8 (17.7, 19.9) | 19% | n/a |
| **High SBP** |  |  |  |  |  |  |  |
| SBP: 140-160mmHg | 3 visits/ year | 1/2 anti-hypertensives | Liver test | $299.6 | M:14.0 (13.1, 15.0)  F: 9.5 (8.7, 10.3) | 40% | 87% |
| SBP: >160 mmHg |  | 3 anti-hypertensives |  | $473.9 | M:2.7 (2.3, 3.2)  F: 5.4 (4.8, 6.0) | 40% | 87% |
| ***High TC** | 1 visits/ year | Atorvastatin | TC & HDL test  Liver test | $261.0 | M:3.3 (2.8, 3.8)  F: 3.0 (2.5, 3.5) | 85% | 70% |
| **^High SBP &TC** | 3 visits/year | 3 anti-hypertensives &  Atorvastatin |  | $682.8 | M:3.6 (3.1, 4.1)  F: 2.0 (1.6, 2.4) | 85% | 70% |

**TC>7.5 mmol/L; ^SBP>140 mmHg &TC >6.5 mmol/L*
