# Supplementary material for: Cost-effectiveness evaluation of the 45-49 year old health check versus usual care in Australian general practice: A modelling study
Source: PLoS One. 2018 Nov 9;13(11):e0207110. doi: 10.1371/journal.pone.0207110 (PMC6226178; doi:10.1371/journal.pone.0207110)
Supplement: S3 Table — (DOCX) [file pone.0207110.s003.docx]

S3 Table: Model validation — Annual CVD incidence (45-54 year old Australians)

| **% (%, %)** | **CHD** | **CVA** | **CVD** |
| --- | --- | --- | --- |
| **Male** | **Mean (2.5, 97.5 percentiles)** | | |
| Observed* | 0.80 | 0.12 | 0.92 |
| Reference model** | 0.810 (0.793, 0.827) | 0.096 (0.094, 0.097) | 0.905 (0.887, 0.923) |
| **Female** | **Mean (2.5, 97.5 percentiles)** | | |
| Observed* | 0.28 | 0.10 | 0.38 |
| Reference model** | 0.320 (0.312, 0.329) | 0.060 (0.059, 0.061) | 0.380(0.371, 0.389) |

**hospitalization episodes in the 45-54 years old Australian population (NHMD); **estimates from 1,000 runs of cohort simulations*
